# Hemilabile Ligands as Mechanosensitive Electrode Contacts for Molecular Electronics†

**DOI:** 10.1002/anie.201906400

**Published:** 2019-08-19

**Authors:** Nicolò Ferri, Norah Algethami, Andrea Vezzoli, Sara Sangtarash, Maeve McLaughlin, Hatef Sadeghi, Colin J. Lambert, Richard J. Nichols, Simon J. Higgins

**Affiliations:** ^1^ Department of Chemistry University of Liverpool Crown Street Liverpool L69 7ZD UK; ^2^ Department of Physics Lancaster University Lancaster LA1 4YB UK; ^3^ School of Engineering University of Warwick Coventry CV4 7AL UK

**Keywords:** density functional calculations, hemilabile ligands, molecular devices, molecular electronics, sulfur ligands

## Abstract

Single‐molecule junctions that are sensitive to compression or elongation are an emerging class of nanoelectromechanical systems (NEMS). Although the molecule–electrode interface can be engineered to impart such functionality, most studies to date rely on poorly defined interactions. We focused on this issue by synthesizing molecular wires designed to have chemically defined hemilabile contacts based on (methylthio)thiophene moieties. We measured their conductance as a function of junction size and observed conductance changes of up to two orders of magnitude as junctions were compressed and stretched. Localised interactions between weakly coordinating thienyl sulfurs and the electrodes are responsible for the observed effect and allow reversible monodentate⇄bidentate contact transitions as the junction is modulated in size. We observed an up to ≈100‐fold sensitivity boost of the (methylthio)thiophene‐terminated molecular wire compared with its non‐hemilabile (methylthio)benzene counterpart and demonstrate a previously unexplored application of hemilabile ligands to molecular electronics.

## Graphical abstract

**Contact request**: Molecular junctions with hemilabile contact moieties show enhanced mechanoresistive behaviour. The molecule–metal contact is forced to transition from a monodentate to a bidentate configuration as the junction is compressed and stretched, with a resulting modulation in conductance of up to two orders of magnitude.

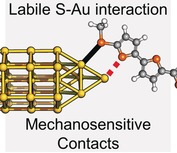

## Introduction

Hemilabile ligands are heterobifunctional ligands in which one donor coordinates strongly to a metal and the other coordinates weakly, often reversibly, as a consequence of the chelate effect. They have found wide applications in homogeneous catalysis,^1^ where they are used, for instance, to reduce the activation energy of steps such as oxidative addition/reductive elimination, and increase reaction rate and turnover, to stabilise reactive intermediates and boost yield values, and to obtain high enantiomeric excesses in catalytic hydrovinylation reactions.^2^ More niche applications can be found in metal‐organic frameworks (MOF) to greatly increase their hydrolytic stability,^3^ and in their use as chemosensors^4^ and redox‐switchable ligands.^5^ The possibility of using external stimuli to change the coordination motif (monodentate–chelate transitions)^6^, ^7^, ^8^ triggered our interest in their development as contacts for molecular electronics, where metallophilic termini (for example, thiols,^9^ amines,^10^ thioethers,^11^ phosphines,^12^ or selenols^13^) are regularly used to chemically and electrically couple the molecular backbone to the nanoelectrodes and fabricate metal–molecule–metal devices. In such junctions, the interface between the molecule and the electrodes is a key parameter for the control of its charge‐transport properties. In addition to introducing an additional (contact) resistance,^11^ the metal–molecule interface controls the extent of coupling between the molecular backbone and the two metallic leads, which can drastically affect the final conductance of the junction.^14^, ^15^

**Figure 1:**
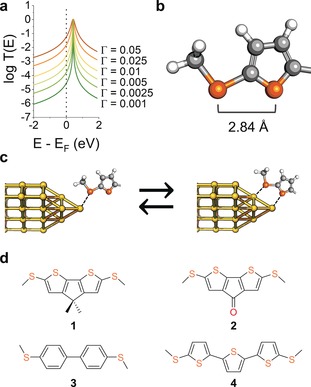
a) Examples of Breit–Wigner resonances for transport through a LUMO of energy *E*_F_+0.5 eV over a range of values of the coupling parameter Γ. No additional energy shift *σ* was used in the calculations for clarity. b) Structure of the hemilabile ligand used in this study, (methylthio)thiophene, and c) examples of its monodentate and bidentate contact configurations at a Au electrode. d) Structures and nomenclature of the compounds used in this study.

The zero‐bias electrical conductance *G* of a molecular electronic device is defined by the Landauer formula,^16^ which takes the form

(1) $$G=G{}_{0}\;T\left(E{}_{F}\right)$$

at low temperature, where *G*_0_ is the quantum of conductance (2 *e* ^2^/*h*≈77.48 μS) and the transmission coefficient *T*(*E*) is the probability for an electron of energy *E* to tunnel through the molecule. The transmission coefficient for a one‐level system with a molecular orbital of energy *ϵ* coupled to the electrodes can be described by the Breit–Wigner formula^17^

(2) $$T\left(E_{F}\right)=\;\frac{4\Gamma^{2}}{{\left(E_{F}-\epsilon -\sigma \right)}^{2}+4\Gamma^{2}}$$

where *σ* is an energy shift of the orbital due to interactions with the electrodes, and Γ describes the strength of the coupling that causes a broadening of the orbital resonance, as shown in Figure 1 a. It can be inferred from the above equations that i) the off‐resonance transmission coefficient (and therefore the conductance of the molecular junction) is strongly dependent on the strength of the coupling to the electrodes, and ii) if Γ can be modulated reversibly and reproducibly in response to an external stimulus, then this would yield a functional nanoelectronic device. We focussed our efforts on the use of mechanical forces as the triggering stimuli, because these have been used to exert some degree of control on Γ by exploiting weak interactions between the metallic electrodes and a neighbouring π‐system in a *hapto* coordination, for instance by using 4‐pyridyl contact groups^18^ or vicinal phenyl rings.^19^, ^20^, ^21^ In the following, we demonstrate that hemilabile ligands also satisfy the above requirements, with further advantages over the existing strategies lying in chemical control on Γ modulation, chemically‐defined contact configurations, and improved sensitivity and amplitude of conductance changes. The reason lies in an enhanced metal–molecule coupling in the bidentate contact compared with the monodentate configuration (Figure 1 c), with a fully reversible mono‐to‐bidentate transition and reproducible behaviour imparted by the hemilabile ligands.

## Results and Discussion

We designed molecular wires with hemilabile contacts, where the strong coordination site is placed at the two termini of the molecule, and compression of the junction would allow the interaction of the metal electrode with a weaker coordinating group, promoting a transition to a bidentate configuration and increasing the coupling parameter Γ. We synthesised molecules terminated with chemically‐defined hemilabile (methylthio)thiophene contacts (Figure 1 b). The methyl thioether substituent acts as the primary contact and creates strong mechanical coupling, while the thienyl moiety interacts less strongly with the metallic electrode (through the S‐atom lone pair), thereby providing the additional electronic coupling to enhance conductance in the compressed junction, with a DFT‐calculated S_thioether_⋅⋅⋅S_thiophene_ separation of 2.84 Å. Thiophenes are known to interact with Au electrodes and have been successfully used as molecular termini in several single‐entity electronics studies.^22^, ^23^, ^24^, ^25^, ^26^, ^27^, ^28^ The interaction is reported as being weaker than traditional contact groups,^29^ consistent with the known coordination chemistry of thiophenes^30^, ^31^, ^32^ and their established use in hemilabile ligand design,^33^, ^34^, ^35^ thus making them an ideal “supporting” molecular contact to the stronger methyl thioether. Furthermore, there are reports in the literature of unusual properties of oligothiophene‐based molecular wires, showing non‐monotonic conductance attenuation with length,^36^, ^37^, ^38^ stretching‐induced conductance decrease,^38^ and large spread of conductance values^39^ (see the Supporting Information for further details). We reasoned these were strong enough evidence in support of our hemilabile contact design, worthy of a more thorough investigation with an electromechanical approach.^21^

**Figure 2:**
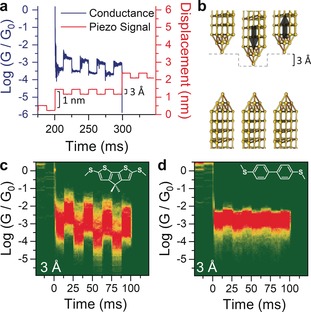
a) Example of a typical 3 Å modulation measurement, with the imposed piezo signal (red) and the corresponding conductance trace (blue) showing the correlation between increase in conductance and junction compression. b) Idealised position of the electrodes during the modulation. c), d) Conductance‐vs.‐time density maps for compound **1** (c) and **3** (d) under square‐wave modulation of 3 Å. The density maps are normalised to the number of scans used to compile them, which is 828 (c) and 980 (d). The structure of the molecular wire is superimposed on the respective density map.

We synthesised the compounds presented in Figure 1 d (for synthetic details and characterization, see the Supporting Information), focusing on the bridged bithiophenes **1**–**2** to reduce the conformational‐isomerism issues arising with conventional oligothiophenes, which result in a large spread of conductance values.^39^ We characterised the electromechanical behaviour of these compounds by employing a modified^18^, ^20^ scanning‐tunnelling‐microscopy break‐junction (*STM‐BJ*) technique.^9^ In a typical experiment, a piezoelectric actuator drives a Au tip into a Au substrate at a fixed bias to form a contact with conductance >5 G_0_. The Au–Au junction is then abruptly stretched by 1 nm and a modulation is applied to the piezo signal for 100 ms (Figure 2 a). The junction is then further stretched and modulated several times to ensure the formation of a single‐molecule junction and its subsequent clean rupture, with an overall stretching of 8 nm. The tip is then driven towards the substrate until the contact once again has a conductance >5 G_0_, and the process is then repeated thousands of times. After data collection, we used an algorithm to slice the traces between abrupt stretches and to discard the traces with no evidence of junction formation and those where the single‐molecule junction did not survive the whole modulation process. On average across the experiment presented here, 38 % of the traces are kept by the algorithm, and these are subsequently compiled into density maps. More details about the measurements and data analysis can be found in the methods section and the Supporting Information. We started our investigation on compound **1** by applying a 3 Å square‐wave modulation to the piezo actuator, which abruptly changes the electrode separation and holds the position for 12.5 ms to allow junction relaxation. The amplitude of 3 Å was chosen to be very close to the S⋅⋅⋅S distance in the (methylthio)thiophene moiety, and therefore a mechanically‐induced monodentate⇄bidentate transition is expected.

**Figure 3:**
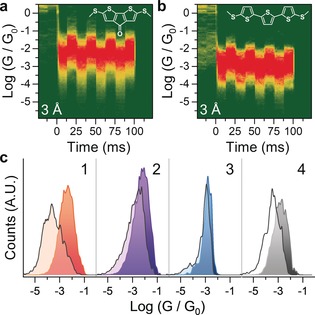
a), b) Conductance‐vs.‐time density maps of compound **2** (a) and **4** (b) under 3 Å modulation. The structure of the molecular wire is superimposed on the respective density map. Density maps are normalised to the number of scans used to compile them, which is 1221 (a) and 1072 (b). c) 1D histograms of the four compounds presented in this study, obtained from the last modulation shown in the density maps by collapsing it on the *x* axis. The compressed junction is reproduced in darker colour, while the extended junction is overlaid in lighter colour.

As can be observed in Figure 2 c, the square‐wave modulation applied to the electrode separation results in large and well‐defined changes in the conductance of **1**. Conductance modulation is reduced when the amplitude of the square wave applied to the piezo actuator is decreased from 3 Å to 2 Å but does not change significantly when the amplitude is increased to 4 Å (See the Supporting Information for density maps). For comparison, the biphenyl‐based compound **3** showed a very small conductance change upon modulation (Figure 2 d), which might be due to increased electrode–electrode direct tunnelling or the already reported increased interactions of the electrodes with the phenyl π‐system as these junctions are compressed (lateral coupling).^19^, ^21^ The striking difference in behaviour between these two simple biaryl compounds suggests that the presence of an additional, labile binding site leads to a larger overall increase of the coupling parameter Γ, as hypothesised earlier. To further characterise this phenomenon and the versatility of (methylthio)thiophene as a hemilabile contact moiety, we turned our attention to compounds **2** and **4**. In compound **2**, the carbonyl substituent has an electron‐withdrawing effect, and we reasoned this would result in reduced thienyl‐S–electrode coupling, thereby decreasing the amplitude of mechanoresistive modulation. Compound **4** is a longer oligothiophene and its purpose is to test whether mechanosensitive properties are retained in longer molecular wires. By using the same 3 Å square‐wave modulation presented earlier, we found, as expected, that molecule **2** shows conductance changes of significantly reduced magnitude compared with **1** (Figure 3 a). Well‐defined conductance variations are also found for **4** (Figure 3 b), although this longer molecule also shows a smaller modulation than **1**. The overall results confirm that the labile behaviour of the thienyl moiety is indeed responsible for the observed behaviour, with **1** being the compound providing the largest conductance variation upon modulation of the electrode position as evidenced by analysing the modulation profile (Figure 3 c). The fact that both conformationally locked molecules **1** and **2**, and conformationally flexible molecule **4** show significant modulation in conductance upon junction compression supports the notion that an explanation for the conductance switching based on possible different conformers^40^ of oligothiophenes^36^ like **4** is not reasonable here.

**Figure 4:**
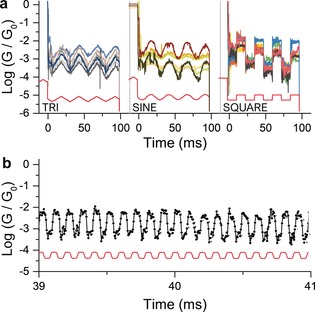
Conductance signal for compound **1** under different types of modulation (a) and under high‐speed (10 kHz) trapezoidal modulation (b). The piezo signal is displayed in red along with the conductance curves. Only a 2 ms portion of a single high‐frequency modulation trace is displayed here for clarity. Additional data can be found in the Supporting Information.

To better characterise the phenomena behind the metal–molecule monodentate⇄bidentate contact transitions, we applied more incremental compression/elongation ramps, which would also allow us to assess the evolution of the coupling parameter Γ as the junction is compressed and relaxed to gain insights into the kinetics of hemilabile coordination. Surprisingly, we did not observe a sudden change in conductance as the junction is slowly compressed or stretched, which would mark a clear monodentate⇄bidentate configuration change. The overall results (see Figure 4 a) suggest that there is no abrupt coordination transition for these compounds but rather an increasing metal–thiophene interaction as the two entities are brought in spatial vicinity, which accounts for the observed increase in conductance. The junctions we fabricated therefore behave more like a nanoelectromechanical rheostat than a simple on/off molecular switch. For example, by slowly compressing (stretching) junctions fabricated with **1** with a triangular ramp at a rate of 24 nm s^−1^, their conductance continuously increases (decreases) to the extent of $e^{\pm 0.78}/\overset{\circ }{A}$ , and similar results were obtained under sinusoidal modulation of the junction size. This absence of a clean coordination change may be of interest for the possible mechanisms of metal‐organic configuration transitions in the fluxional behaviour in solution of thienyl‐based^41^, ^42^ and other weak‐link hemilabile ligands,^43^, ^44^, ^45^ although other factors will be of importance in solution reactions, such as solvation energies and related factors.

We also performed high‐frequency modulation experiments (10 kHz, Figure 4 b), and compound **1** showed regular changes in conductance correlating with the signal imposed to the piezo actuator (additional details are given in the Supporting Information), with no apparent fatigue for up to 1000 modulations. It should be noted that a 10 kHz frequency is at our instrument limit (200 kSa s^−1^ data acquisition system, resulting in 20 points per modulation cycle) and it is therefore possible that even higher frequencies can be attained.

**Figure 5:**
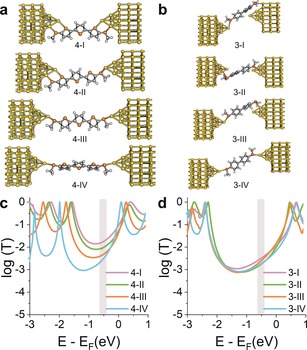
a), b) Relaxed structures of molecule **4** (a) and **3** (b) between two Au electrodes at various tip–tip distances. Starting separation (**4‐I**: 11.9, **3‐I**: 8.8 Å) increased by approximately 2 Å at each step. Colour coding: H=white, C=grey, S=orange, Au=yellow. c), d) Corresponding transmission coefficient *T*(*E*) vs. electron energy of compound **4** (c) and **3** (d) at the four tip–tip distances. *E* is plotted relative to the DFT‐predicted Fermi energy of gold. The grey region shows the window of Fermi‐energy values where the amplitude of conductance is in good agreement with the experimentally measured values.

To better understand the phenomena leading to the observed changes in conductance, and thereby to quantify the effect of variations of Γ, we used density functional theory (DFT) to compute the quantum transport properties for all the molecules (see the Supporting Information for details). The quantum‐transport code Gollum^46^ was used to calculate the transmission coefficient *T*(*E*) for electrons of energy *E* passing from the source to the drain electrode via the molecule.^47^ Using compound **4** as an example (Figure 5 a), we predict that at small tip–tip distances, the gold electrodes interact with both thienyl and thioether sulfurs, resulting in high molecule–electrode coupling and a broadening of transport resonances (Figure 5 c). As the electrode separation is increased, the coupling to the thiophene moiety is reduced and the resonance becomes sharper. No such effects are found in **3** (Figure 5 b,d). It is worth noting that when there is a change in the degree of molecule–electrode Au–S interaction, the position of the energy levels relative to *E*_F_ changes as well. This is due to different degrees of charge transfer, which lead to shifts of the whole transmission spectrum.

To predict the effect on the conductance *G*=*G*_0_ *T*(*E*_F_), a value for *E*_F_ is needed. In Figure 5 c,d, the HOMO and LUMO levels correspond to the resonances in the transmission plots *T*(*E*) located immediately below and above *E*−*E*_F_=0 respectively. The precise values of the HOMO and LUMO levels relative to *E*_F_ (and therefore the exact zero of the horizontal axis) depend on environmental conditions and on the unknown shape of the electrodes. However, since the molecules are neither oxidised nor reduced, *E*_F_ lies within the energy gap between the HOMO and the LUMO, and the qualitative change in the conductance with tip–tip distance is determined by the behaviour of *T*(*E*) within the gap. In compound **4,** the additional thiophene–Au interactions described earlier result in broadened transport resonances with an accompanying increase of the mid‐gap value of *T*(*E*) as the junction is compressed, and therefore the conductance is also expected to increase. In compound **3**, which lacks a thiophene moiety, the resonances stay sharp as the junction is compressed and value of *T*(*E*) within the gap does not change significantly. Therefore, the conductance is predicted to be almost independent of the electrode separation. The process was repeated for compounds **1** and **2** (details in the Supporting Information) and, in agreement with our experiments, compressing the electrodes leads to strong interactions between the electrodes and the thiophene moieties, causing significant variations of the transmission coefficient over a wide range of energies within the HOMO–LUMO gap. Furthermore, for values of *E*_F_ lying in the window highlighted as a grey box in Figure 5 c,d, there is good agreement with the experimental values shown in Figure 3 c, with compound **1** having the largest variations in *T*(*E*) as the electrode separation is modulated, and compound **3** showing little or no effect. By fitting the Breit–Wigner formula to the LUMO resonance of the *T*(*E*) curves for the four compounds presented in this study, we found a pattern of increasing values of Γ for compounds **1, 2**, and **4** as the junction is compressed, but Γ for compound **3** does not change significantly over a range of several Å of junction‐size modulation (see the Supporting Information for more details). As further confirmation of the proposed mechanism, the coupling parameters for compound **3** were found to be consistently lower than for compound **1** at similar junction size, in agreement with the reduced conductance modulation observed during junction compression and elongation.

## Conclusion

In conclusion, we have shown how hemilabile ligands, in this case based on the (methylthio)thiophene moiety, can be exploited to impart mechanosensitive behaviour to a single‐molecule junction. The presence of multiple binding sites and the monodentate⇄bidentate contact transition was exploited to modulate the coupling parameter Γ as the junction is compressed and relaxed. The junctions displayed chemically controlled sensitivity, can operate at kHz speed and show no sign of fatigue for up to 1000 cycles. In addition to expanding the molecular‐electronics toolbox with new design ideas for molecular termini and contacts, our results shed light on the mechanism of configuration transitions in thienyl‐based hemilabile ligands, showing that the fabrication of single‐molecule junctions doubles also as a valuable characterisation tool to probe metal–ligand interactions. Furthermore, (methylthio)thiophenes and thiophenethiols are widely used as molecular‐wire termini in molecular electronics,^36^, ^39^, ^48^, ^49^, ^50^, ^51^, ^52^, ^53^, ^54^ and our results unambiguously demonstrate that contact‐configuration changes must be taken into account when interpreting their conductance‐vs.‐electrode‐separation trajectories during a single‐entity break‐junction experiment.

## Experimental Section

**Synthesis of compounds 1**–**4**: Compound **1** was synthesised by methylation of commercial 4*H*‐cyclopenta[2,1‐*b*:3,4‐*b′*]dithiophene with methyl iodide and potassium hydroxide, followed by selective bromination with NBS, halogen‐lithium exchange with *n*‐butyllithium and quenching with dimethyl disulfide. Compound **2** was prepared from commercial 2,2′‐bithiophene by tetrabromination with elemental bromine, followed by halogen–lithium exchange with *n*‐butyllithium and quenching with dimethyl disulfide to yield 3,3′‐dibromo‐5,5′‐bis(thiomethyl)‐2,2′‐bithiophene. Halogen–lithium exchange with *n*‐butyllithium, followed by low‐temperature quenching with dimethylcarbamoyl chloride gave **2**. Compound **3** was prepared by halogen–lithium exchange of 4,4′‐diiodo‐1,1′‐biphenyl with *n*‐butyllithium, followed by quenching with dimethyl disulfide. Compound **4** was prepared by double direct lithiation of 2,2′:5′,2′′‐terthiophene with *n*‐butyllithium, followed by quenching with dimethyl disulfide.

**Scanning‐Tunneling‐Microscope Break Junction**: Molecular junctions are formed by repeatedly driving a Au tip into a Au substrate and then pulling it apart in a solution of the desired molecular wire (mesitylene, 1 mm). A constant bias (200 mV) is applied between the tip and the substrate during the measurements, which are performed at room temperature. The tip is retracted in a stepwise manner, creating a nanogap which is held for 100 ms while its size is modulated (see Figure 2 a for the signal applied to the piezoelectric actuator). The tip is then retracted until junction rupture and driven into the substrate again. The process is continuously repeated. 1000 cycles are performed on the bare Au substrate prior to the measurements, to perform a mechanical annealing of the tip and ensure absence of contaminants. After the introduction of the molecular wire, 2000–3000 scans are collected and then analyzed using automated algorithms.

**DFT calculations**: The optimised geometry, ground‐state Hamiltonian, and overlap‐matrix elements of each structure were obtained self‐consistently using DFT and the SIESTA code.^55^ SIESTA employs norm‐conserving pseudo‐potentials to account for the core electrons and linear combinations of atomic orbitals to construct the valence states. The PBE functional, a DZP basis set, and a real‐space grid defined with an equivalent energy cutoff of 250 Ry were used. The geometry optimisation for each structure was performed until forces were below 10 meV Å^−1^.

**Transport calculations**: The mean‐field Hamiltonian obtained from the converged DFT calculation was combined with the Gollum^46^ implementation of the non‐equilibrium Green's function method to calculate the phase‐coherent elastic‐scattering properties of each system consisting of left gold (source) and right gold (drain) leads and the scattering region.

Supporting Information available: Synthetic procedures and characterization data, further details on the STM‐BJ experiments, equipment used, measurement procedure and data analysis algorithms, and further theoretical calculations. NMR spectra (Bruker format) for compounds **1**–**4** and the raw STM‐BJ data can be found in the data catalogue in Liverpool at https://datacat.liverpool.ac.uk/id/eprint/549 and at https://doi.org/10.17638/datacat.liverpool.ac.uk/549.

## Conflict of interest

The authors declare no conflict of interest.

## Supporting information

As a service to our authors and readers, this journal provides supporting information supplied by the authors. Such materials are peer reviewed and may be re‐organized for online delivery, but are not copy‐edited or typeset. Technical support issues arising from supporting information (other than missing files) should be addressed to the authors.

Supplementary

## References

[1] A. Bader , E. Lindner , Coord. Chem. Rev. 1991, 108, 27–110.

[2] T. V. RajanBabu , Chem. Rev. 2003, 103, 2845–2860. 12914483 10.1021/cr020040g

[3] L. N. McHugh , M. J. McPherson , L. J. McCormick , S. A. Morris , P. S. Wheatley , S. J. Teat , D. McKay , D. M. Dawson , C. E. F. Sansome , S. E. Ashbrook , et al., Nat. Chem. 2018, 10, 1096–1102. 30104722 10.1038/s41557-018-0104-x

[4] S. E. Angell , C. W. Rogers , Y. Zhang , M. O. Wolf , W. E. Jones , Coord. Chem. Rev. 2006, 250, 1829–1841.

[5] H. F. Cheng , A. I. D'Aquino , J. Barroso-Flores , C. A. Mirkin , J. Am. Chem. Soc. 2018, 140, 14590–14594. 30365302 10.1021/jacs.8b09321

[6] A. J. M. Miller , Dalton Trans. 2017, 46, 11987–12000. 28852761 10.1039/c7dt02156a

[7] A. M. Lifschitz , C. M. Shade , A. M. Spokoyny , J. Mendez-Arroyo , C. L. Stern , A. A. Sarjeant , C. A. Mirkin , Inorg. Chem. 2013, 52, 5484–5492. 23570551 10.1021/ic400383t

[8] A. M. Allgeier , C. S. Slone , C. A. Mirkin , L. M. Liable-Sands , G. P. A. Yap , A. L. Rheingold , J. Am. Chem. Soc. 1997, 119, 550–559.

[9] B. Xu , N. Tao , Science 2003, 301, 1221–1223. 12947193 10.1126/science.1087481

[10] Y. Zang , A. Pinkard , Z.-F. Liu , J. B. Neaton , M. L. Steigerwald , X. Roy , L. Venkataraman , J. Am. Chem. Soc. 2017, 139, 14845–14848. 28981277 10.1021/jacs.7b08370

[11] P. Moreno-García , M. Gulcur , D. Z. Manrique , T. Pope , W. Hong , V. Kaliginedi , C. Huang , A. S. Batsanov , M. R. Bryce , C. Lambert , et al., J. Am. Chem. Soc. 2013, 135, 12228–12240. 23875671 10.1021/ja4015293

[12] Y. S. Park , A. C. Whalley , M. Kamenetska , M. L. Steigerwald , M. S. Hybertsen , C. Nuckolls , L. Venkataraman , J. Am. Chem. Soc. 2007, 129, 15768–15769. 18052282 10.1021/ja0773857

[13] S. Yasuda , S. Yoshida , J. Sasaki , Y. Okutsu , T. Nakamura , A. Taninaka , O. Takeuchi , H. Shigekawa , J. Am. Chem. Soc. 2006, 128, 7746–7747. 16771482 10.1021/ja062066l

[14] C. J. Lambert , Chem. Soc. Rev. 2015, 44, 875–888. 25255961 10.1039/c4cs00203b

[15] M. Koch , Z. Li , C. Nacci , T. Kumagai , I. Franco , L. Grill , Phys. Rev. Lett. 2018, 121, 47701. 10.1103/PhysRevLett.121.047701 30095964

[16] R. Landauer , IBM J. Res. Dev. 1957, 1, 223–231.

[17] G. Breit , E. Wigner , Phys. Rev. 1936, 49, 519–531.

[18] S. Y. Quek , M. Kamenetska , M. L. Steigerwald , H. J. Choi , S. G. Louie , M. S. Hybertsen , J. B. Neaton , L. Venkataraman , Nat. Nanotechnol. 2009, 4, 230–234. 19350032 10.1038/nnano.2009.10

[19] I. Diez-Perez , J. Hihath , T. Hines , Z.-S. Wang , G. Zhou , K. Müllen , N. Tao , Nat. Nanotechnol. 2011, 6, 226–231. 21336268 10.1038/nnano.2011.20

[20] A. K. Ismael , K. Wang , A. Vezzoli , M. K. Al-Khaykanee , H. E. Gallagher , I. M. Grace , C. J. Lambert , B. Xu , R. J. Nichols , S. J. Higgins , Angew. Chem. Int. Ed. 2017, 56, 15378–15382; 10.1002/anie.201709419 29044889

[21] R. Ramachandran , H. B. Li , W.-Y. Lo , A. Neshchadin , L. Yu , J. Hihath , Nano Lett. 2018, 18, 6638–6644. 30247037 10.1021/acs.nanolett.8b03415

[22] M. Kiguchi , T. Ohto , S. Fujii , K. Sugiyasu , S. Nakajima , M. Takeuchi , H. Nakamura , J. Am. Chem. Soc. 2014, 136, 7327–7332. 24624980 10.1021/ja413104g

[23] R. Frisenda , R. Gaudenzi , C. Franco , M. Mas-Torrent , C. Rovira , J. Veciana , I. Alcon , S. T. Bromley , E. Burzurí , H. S. J. van der Zant , Nano Lett. 2015, 15, 3109–3114. 25897770 10.1021/acs.nanolett.5b00155

[24] J.-C. Mao , L.-L. Peng , W.-Q. Li , F. Chen , H.-G. Wang , Y. Shao , X.-S. Zhou , X. Zhao , H.-J. Xie , Z. Niu , J. Phys. Chem. C 2017, 121, 1472–1476.

[25] A. Etcheverry-Berríos , I. Olavarría , M. L. Perrin , R. Díaz-Torres , D. Jullian , I. Ponce , J. H. Zagal , J. Pavez , S. O. Vásquez , H. S. J. van der Zant , et al., Chem. Eur. J. 2016, 22, 12808–12818. 27458818 10.1002/chem.201601187

[26] C. R. Arroyo , S. Tarkuc , R. Frisenda , J. S. Seldenthuis , C. H. M. Woerde , R. Eelkema , F. C. Grozema , H. S. J. van der Zant , Angew. Chem. Int. Ed. 2013, 52, 3152–3155; 10.1002/anie.201207667 23386366

[27] S. Bock , O. A. Al-Owaedi , S. G. Eaves , D. C. Milan , M. Lemmer , B. W. Skelton , H. M. Osorio , R. J. Nichols , S. J. Higgins , P. Cea , et al., Chem. Eur. J. 2017, 23, 2133–2143. 27897344 10.1002/chem.201604565 PMC5396322

[28] Y. Ie , K. Tanaka , A. Tashiro , S. K. Lee , H. R. Testai , R. Yamada , H. Tada , Y. Aso , J. Phys. Chem. Lett. 2015, 6, 3754–3759. 26722752 10.1021/acs.jpclett.5b01662

[29] F. Bejarano , I. J. Olavarria-Contreras , A. Droghetti , I. Rungger , A. Rudnev , D. Gutiérrez , M. Mas-Torrent , J. Veciana , H. S. J. van der Zant , C. Rovira , et al., J. Am. Chem. Soc. 2018, 140, 1691–1696. 29307191 10.1021/jacs.7b10019

[30] J. Chen , R. J. Angelici , Coord. Chem. Rev. 2000, 206–207, 63–99.

[31] R. J. Angelici , Organometallics 2001, 20, 1259–1275.

[32] T. Jiang , W. Malone , Y. Tong , D. Dragoe , A. Bendounan , A. Kara , V. A. Esaulov , J. Phys. Chem. C 2017, 121, 27923–27935.

[33] D. A. Weinberger , T. B. Higgins , C. A. Mirkin , C. L. Stern , L. M. Liable-Sands , A. L. Rheingold , J. Am. Chem. Soc. 2001, 123, 2503–2516. 11456918 10.1021/ja0030008

[34] O. Clot , M. O. Wolf , G. P. A. Yap , B. O. Patrick , J. Chem. Soc. Dalton Trans. 2000, 2729–2737.

[35] D. A. Weinberger , T. B. Higgins , C. A. Mirkin , L. M. Liable-Sands , A. L. Rheingold , Angew. Chem. Int. Ed. 1999, 38, 2565–2568; 10.1002/(sici)1521-3773(19990903)38:17<2565::aid-anie2565>3.0.co;2-u 10508340

[36] B. Capozzi , E. J. Dell , T. C. Berkelbach , D. R. Reichman , L. Venkataraman , L. M. Campos , J. Am. Chem. Soc. 2014, 136, 10486–10492. 25003761 10.1021/ja505277z

[37] L. Xiang , T. Hines , J. L. Palma , X. Lu , V. Mujica , M. A. Ratner , G. Zhou , N. Tao , J. Am. Chem. Soc. 2016, 138, 679–687. 26694660 10.1021/jacs.5b11605

[38] B. Q. Xu , X. L. Li , X. Y. Xiao , H. Sakaguchi , N. J. Tao , Nano Lett. 2005, 5, 1491–1495. 16178263 10.1021/nl050860j

[39] E. J. Dell , B. Capozzi , K. H. DuBay , T. C. Berkelbach , J. R. Moreno , D. R. Reichman , L. Venkataraman , L. M. Campos , J. Am. Chem. Soc. 2013, 135, 11724–11727. 23905714 10.1021/ja4055367

[40] L. Mejía , N. Renaud , I. Franco , J. Phys. Chem. Lett. 2018, 9, 745–750. 29369638 10.1021/acs.jpclett.7b03323

[41] S. Liu , C. R. Lucas , M. J. Newlands , J. P. Charland , Inorg. Chem. 1990, 29, 4380–4385.

[42] G. R. Haire , N. E. Leadbeater , J. Lewis , P. R. Raithby , A. J. Edwards , E. C. Constable , J. Chem. Soc. Dalton Trans. 1997, 2997–3004.

[43] F. E. Kühn , W. A. Herrmann , M. Cokoja , A. Raba , A. Pöthig , K. Riener , M. J. Bitzer , Inorg. Chem. 2014, 53, 12767–12777. 25423584 10.1021/ic5016324

[44] G. M. Adams , A. S. Weller , Coord. Chem. Rev. 2018, 355, 150–172.

[45] E. Lindner , M. Haustein , H. A. Mayer , H. Kühbauch , K. Vrieze , B. de Klerk-Engels , Inorg. Chim. Acta 1994, 215, 165–172.

[46] J. Ferrer , C. J. Lambert , V. M. García-Suárez , D. Z. Manrique , D. Visontai , L. Oroszlany , R. Rodríguez-Ferradás , I. Grace , S. W. D. Bailey , K. Gillemot , et al., New J. Phys. 2014, 16, 093029.

[47] H. Sadeghi , Nanotechnology 2018, 29, 373001. 29926808 10.1088/1361-6528/aace21

[48] M. Tateno , M. Takase , T. Nishinaga , Chem. Mater. 2014, 26, 3804–3810.

[49] G. Gryn'ova , P. J. Ollitrault , C. Corminboeuf , Phys. Chem. Chem. Phys. 2017, 19, 23254–23259. 28825751 10.1039/c7cp04295g

[50] W. B. Chang , C.-K. Mai , M. Kotiuga , J. B. Neaton , G. C. Bazan , R. A. Segalman , Chem. Mater. 2014, 26, 7229–7235.

[51] Z. Cai , W.-Y. Lo , T. Zheng , L. Li , N. Zhang , Y. Hu , L. Yu , J. Am. Chem. Soc. 2016, 138, 10630–10635. 27488536 10.1021/jacs.6b05983

[52] D. Dulić , F. Pump , S. Campidelli , P. Lavie , G. Cuniberti , A. Filoramo , Angew. Chem. Int. Ed. 2009, 48, 8273–8276; 10.1002/anie.200902168 19728349

[53] C. Kergueris , J. P. Bourgoin , D. Esteve , C. Urbina , M. Magoga , C. Joachim , Phys. Rev. B 1999, 59, 12505–12513.

[54] D. Dulić , S. van der Molen , T. Kudernac , H. Jonkman , J. de Jong , T. Bowden , J. van Esch , B. Feringa , B. van Wees , Phys. Rev. Lett. 2003, 91, 207402. 14683393 10.1103/PhysRevLett.91.207402

[55] J. M. Soler , E. Artacho , J. D. Gale , A. García , J. Junquera , P. Ordejón , D. Sánchez-Portal , J. Phys. Condens. Matter 2002, 14, 2745–2779. 10.1088/0953-8984/20/6/064208 21693870

