# Supplementary material for: Hemilabile Ligands as Mechanosensitive Electrode Contacts for Molecular Electronics
Source: Angew Chem Int Ed Engl. 2019 Aug 19;58(46):16583–9. doi: 10.1002/anie.201906400 (PMC6899542; doi:10.1002/anie.201906400)
Supplement: Supplementary file 1 — Supplementary [file ANIE-58-16583-s001.pdf]

Angewandte Chemie
Eine Zeitschrift der Gesellschaft Deutscher Chemiker

Supporting Information

Hemilabile Ligands as Mechanosensitive Electrode Contacts for Molecular Electronics**

Nicolò Ferri+, Norah Algethami+, Andrea Vezzoli+, Sara Sangtarash+,* Maeve McLaughlin, Hatef Sadeghi, Colin J. Lambert,* Richard J. Nichols, and Simon J. Higgins**

anie_201906400_sm_miscellaneous_information.pdf

Supporting Information

Table of Contents

**1. Synthetic details**	**S2**
**2. Details on STM-BJ measurements**	**S7**
**3. Additional single-molecule conductance data**	**S11**
**4. Details on DFT methods and additional calculations**	**S18**
**5. References**	**S21**

1. Synthetic Details

All reactions were performed under inert gas (Ar) in oven-dried glassware and dry solvents. All reagents were purchased from Sigma-Aldrich Chemical Company (now MilliporeSigma) except where otherwise stated, and solvents were purchased from Fisher ThermoScientific. Starting materials, catalysts and solvents were use as received except the following:

- Tetrahydrofuran (THF) was dried and stored over activated 3 Å molecular sieves (20 to 25 % m/v).[1]
- Alkyllithium reagents were titrated against benzylbenzamide to a blue endpoint before use.[2]
- *N*-bromosuccinimide was recrystallised from milliQ water.

Thin layer chromatography was performed on Merck Silica Gel 60 F-254 plates. Flash column chromatography was performed using Sigma-Aldrich technical grade silica (230-400 mesh, pore size 60 Å), using compressed air. ^{1}H and ^{13}C NMR spectra were recorded on a Bruker Avance 400 Ultrashield spectrometer and referenced to internal TMS and the residual solvent peak. Mass spectra were recorded using an Agilent Q-TOF 7200, and CNH(S) microanalysis was performed with an Elementar Vario MICRO cube.

Compound **1** in the main text was synthesised by methylating commercial 4*H*-cyclopenta[*2,1-b:3,4-b'*]dithiophene (TCI) with methyl iodide and potassium hydroxide, followed by selective bromination with NBS, halogen-lithium exchange with *n*-butyllithium and quench with dimethyl disulfide (Figure S1)

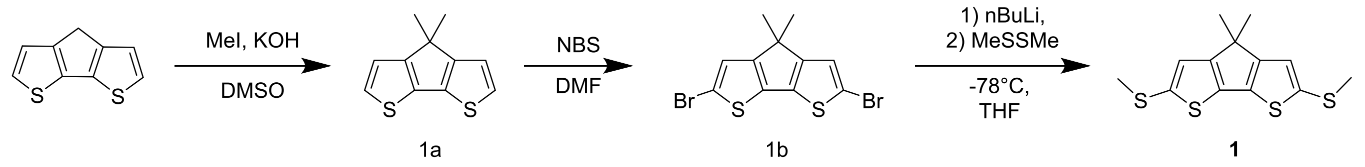

Figure S1: Synthetic pathway to compound **1**

Compound **2** was prepared from commercial 2,2'-bithiophene (Fluorochem) by tetrabromination with elemental bromine (Fisher), followed by halogen-lithium exchange with *n*-butyllithium and quench with dimethyl disulfide to yield 3,3'-dibromo-5,5'-dithiomethyl-2,2'-bithiophene. Halogen-lithium exchange with *n*-butyllithium, followed by low-temperature quench with dimethylcarbamoyl chloride gave **2** (Figure S2).

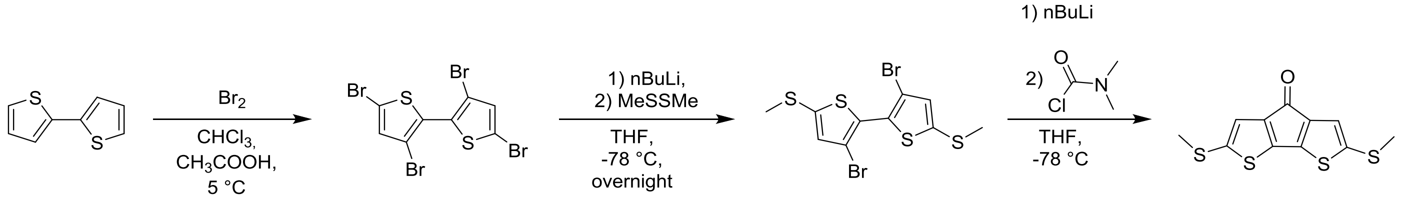

2a 2b **2**

Figure S2: Synthetic pathway to compound **2**.

Compound **3** was prepared by halogen-lithium exchange of 4,4'-diiodobiphenyl (TCI) with *n*-butyllithium, followed by quench with dimethyl disulfide.

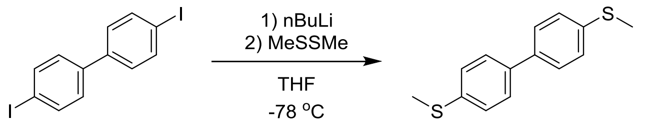

3

Figure S3: Preparation of **3**.

Compound **4** was prepared by double lithiation of 2,2':5',2"-terthiophene,[3] followed by quenching with dimethyl disulfide.

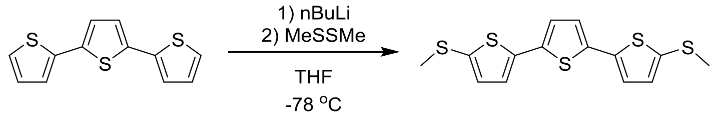

Figure S4: Preparation of **4**.

Preparation of 1a

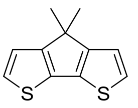

Prepared by modifying a published procedure.[4] A suspension of finely ground potassium hydroxide (1.57 g, 28 mmol) and 4*H*-cyclopenta[2,1-b;3,4-b']dithiophene (1 g, 5.6 mmol) in DMSO (20 mL) was stirred for 1 hour. After this time, methyl iodide (2.39 g, 16 mmol) was added, and the reaction mixture was stirred overnight at room temperature. Water (20 mL) was added to the mixture, and the product was extracted with CH_2Cl_2 (3 x 20 mL). Purification by column chromatography on silica (100 % hexanes) gave the title compound as a white solid (1.10 g, 95 %). ^{1}H NMR (400 MHz, $CDCl_3$) δ:7.07 (d, 2H, J = 4.8 Hz), 6.91 (d, 2H, J = 4.8 Hz), 1.43 (s, 6H). ^{13}C NMR (100 MHz, $CDCl_3$) δ 160.6, 135.2, 124.8, 120.9, 41.0, 25.1.

Preparation of 1b

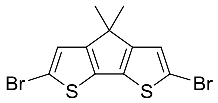

Prepared by modifying a published procedure.[5] To a solution of **1a** (0.5 g, 2.42 mmol) in anhydrous DMF (18 mL), *N*-bromosuccinimide (0.863 g, 4.85 mmol) was added portionwise in the dark, and the reaction mixture was stirred at room temperature for 1 hour under Ar atmosphere. After this time, the suspension was poured into 10 mL of saturated aqueous sodium thiosulfate, and the product extracted with hexane (3 x 20 mL). The combined organic phases were washed with brine, dried over $MgSO_4$, filtered, and the solvent was removed *in vacuo*. Purification by column chromatography on silica (100 % hexanes) and subsequent recrystallisation from boiling hexane gave the title compound as a white solid (0.397 g, 45 %). ^{1}H NMR (400 MHz, $CDCl_3$) δ:7.07 (d, 2H, J = 4.8 Hz), 1.40 (s, 6H). ^{13}C NMR (100 MHz, $CDCl_3$) δ 158.3, 135.1, 124.0, 111.4, 46.4, 24.8.

Preparation of 1

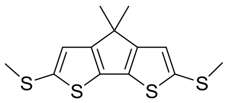

To a solution of **1b** (247 mg, 0.68 mmol) in dry THF (18 mL) under Ar atmosphere that was cooled at -78 °C, *n*-butyllithium in hexane (caution! 1.61 M, 0.93 mL, 1.49 mmol) was added dropwise while stirring. The resulting yellow solution was stirred for 45 minutes before the dropwise addition of dimethyl disulfide (0.13 mL, 1.49 mmol). The mixture was then allowed to reach room temperature and it was stirred overnight. The solvent was then removed *in vacuo* and the crude solid was taken up in CH_2Cl_2 (25 mL) and washed with water (2x10 ml) and brine (15 mL). The organic layer was then dried over $MgSO_4$, filtered, and the solvent was evaporated *in vacuo* to give an off-white solid. Purification by column chromatography on silica (100 % hexanes) and subsequent recrystallization from boiling methanol afforded the title compound as white needle-like crystals (0.095 g, 47 %). Found C = 52.42, H = 4.79, S = 42.46 %. $C_{13}H_{14}S_4$ requires C = 52.31, H = 4.73, S = 42.96 %. ^{1}H NMR (400 MHz, $CDCl_3$) δ:7.27 (s, 2H), 2.50 (s, 6H), 1.41 (s, 6H). ^{13}C NMR (100 MHz, $CDCl_3$) δ 159.54, 137.90, 136.90, 126.49, 45.76, 25,03, 23.37. *m/z* (HRMS, EI, EtOH) 297.9964 $[M+H]^+$. $C_{13}H_{14}S_4$ calc. 297.9978.

Preparation of 2a

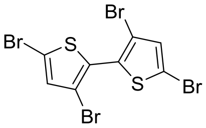

A solution of 2,2'-bithiophene (10.0 g, 60.2 mmol) in acetic acid (50 mL) and chloroform (100 mL) was cooled to 0 °C, and bromine (caution! 16 mL, 300 mmol) was added dropwise over 1 hour. The mixture was then allowed to return to room temperature and it was stirred for 2 hours, followed by warming to 60°C for 2 hours. The mixture was then poured onto 300 mL of ice-cooled methanol and filtered. The residue was recrystallized four times from boiling ethanol to afford the title compound as off-white solid (14.2 g, 49 %). ^{1}H NMR (400 MHz, $CDCl_3$) δ:7.05 (s, 2H). ^{13}C NMR (100 MHz, $CDCl_3$) δ 133.00, 129.55, 114.83, 112.13.

Preparation of 2b

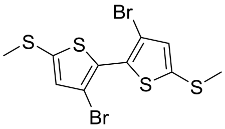

To a solution of **2a** (4.22 g, 8.8 mmol) in dry THF (115 mL) under Ar atmosphere that was cooled at -78 °C, *n*-butyllithium in hexane (caution! 1.58 M, 11mL, 17.5 mmol) was added dropwise while stirring. The resulting yellow solution was stirred for 45 minutes and dimethyl disulfide (1.7 mL, 18.4 mmol) was added dropwise. The mixture was allowed to reach room temperature and it was stirred overnight. The solvent was then removed *in vacuo*, the resulting oil was picked up in CH_2Cl_2 (50 mL) and washed with water (2 x 25 ml) and brine (25 mL). The organic layer was then dried over $MgSO_4$, filtered, and the solvent evaporated *in vacuo* to give an orange oil that was purified by recrystallisation from 1:1 CH_2Cl_2:hexanes at -61 °C to give the title compound as pale-yellow solid (2.84 g, 77 %). ^{1}H NMR (400 MHz, $CDCl_3$) δ: 6.97 (s, 2H), 2.54 (s, 6H). ^{13}C NMR (100 MHz, $CDCl_3$) δ: 140.32, 132.28, 129.62, 111.64, 20.95.

Preparation of 2

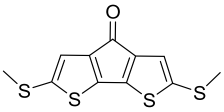

To a solution of **2b** (600 mg, 1.44 mmol) in dry THF (80 mL) under Ar atmosphere that was cooled at -78 °C, *n*-butyllithium in hexane (caution! 1.59 M, 1.8 mL, 2.9 mmol) was added dropwise while stirring. The resulting dark yellow solution was stirred for 30 minutes and a solution of carbamoyl chloride (0.14 mL, 1.44 mmol) in THF (1 mL)

was then added dropwise. The mixture was left stirring at -78 °C for 2.5 hours, and after this time it was warmed at -40 °C and a saturated aqueous solution of NH_4Cl (10 mL) was added. The resulting suspension was allowed to return to room temperature whilst stirring overnight. The organic layer was then separated, and the aqueous layer was extracted once with hexanes (20 mL). The combined organic layers were dried over $MgSO_4$, filtered, and the solvent was removed *in vacuo* to give a dark brown oil that was purified by column chromatography on silica (2 % EtOAc in hexanes) to give a dark purple solid. CHNS microanalysis was not satisfactory, so the solid was dissolved in CH_2Cl_2 and hexanes were carefully layered on top of the solution. Dark violet block crystals grew overnight (330 mg, 80 %). Found: C = 47.26, H = 3.17, S = 45.00 %. $C_{11}H_8OS_4$ requires C = 46.45, H = 2.84, S = 45.09 %. ^{1}H NMR (400 MHz, $CDCl_3$) δ: 7.02 (s, 2H), 2.48 (s, 6H). ^{13}C NMR (100 MHz, $CDCl_3$) δ: 182.25, 150.88, 140.89, 140.54, 126.64, 23.03. *m/z* (HRMS, CI, CH_4) 284.9530 $[M+H]^+$. $C_{11}H_8OS_4$ calc. 283.9458.

Preparation of 3

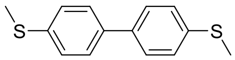

To a solution of 4,4'-diiodobiphenyl (1 g, 2.46 mmol) in THF (45 mL) that was cooled at -78 °C, *n*-butyllithium in hexane (caution! 1.30 M, 3.8 mL, 4.92 mmol) was added dropwise. The resulting milky white suspension was stirred for 30 minutes, and then dimethyl disulfide (0.486 g, 5.16 mmol) was added dropwise at -78 °C. The solution returned clear, and it was stirred at room temperature for 3 hours. After this time, water (40 mL) was added dropwise, and the layers were separated. The aqueous layer was extracted with chloroform (3 x 30 mL), the combined organics were washed with brine (3 x 15 mL), dried with $MgSO_4$ and filtered. After removal of the solvent *in vacuo*, the resulting solid was recrystallised from boiling ethanol to give the title compound as white reflective plates (0.147 g, 25 %). Found: C = 68.22, H = 5.67, S = 26.11 %. $C_{15}H_{15}S_2$ requires C = 68.25, H = 5.73, S = 26.02 %. ^{1}H NMR (400 MHz, $CDCl_3$) δ: 7.51 (dt, J = 8.4, 2.2, 4H), 7.32 (dt, J = 8.4, 2.2, 4H), 2.52 (s, 6H). ^{13}C NMR (100 MHz, $CDCl_3$) δ: 137.7, 137.3, 127.5, 127.1, 16.2. *m/z* (HRMS, CI, CH_4) 247.0612 $[M+H]^+$. $C_{14}H_{15}S_2$ calc. 247.0615.

Preparation of 4

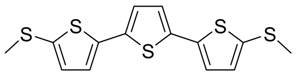

A solution of 2,2':5',2"-terthiophene (0.5 g, 2.01 mmol) in dry THF (40 mL) was cooled to -78 ºC and *n*-butyllithium (caution! 1.55 M, 2.6 mL, 4.00 mmol) was added dropwise whilst stirring. The yellow suspension was stirred for 30 minutes at that temperature and dimethyl disulfide (0.36 mL, 4.05 mmol) was added dropwise. The mixture was

allowed to return to room temperature and was then stirred for 4 hours. Water (100 mL) and CH_2Cl_2 (30 mL) were added, the phases were separated, and the aqueous phase was extracted with CH_2Cl_2 (3 x 25 mL). The combined organic phases were then washed with brine, dried over $MgSO_4$ and concentrated under vacuum. The resulting yellow crude product was then purified by column chromatography (20 % CH_2Cl_2 in hexanes) to afford a slightly impure (NMR) yellow solid. Recrystallisation from hot hexanes afforded the title compound as a bright yellow solid (0.329 g, 48 %). Found: C = 49.34, H = 3.53, S = 48.71 %. $C_{14}H_{12}S_5$ requires C = 49.37, H = 3.55, S = 47.08 %. ^{1}H NMR (400 MHz, $CDCl_3$): 7.01 (s, 2H), 7.00 (d, J = 3.8 Hz, 2H), 6.97 (d, J = 3.8 Hz, 2H), 2.51 (s, 6H) ppm. ^{13}C NMR (100 MHz, $CDCl_3$): 139.0, 136.6, 135.9, 131.8, 124.3, 123.7, 22.1 ppm. *m/z* (HRMS, CI, CH_4) 340.9632 $[M + H]^+$, $C_{14}H_{13}S_5$ calc. 340.9621.

2. Details on *STM-BJ* measurements

Au substrates for *STM-BJ* measurements were purchased from Arrandee Metal GmbH. Substrates were cleaned with ethanol and milliQ water, and annealed with a butane microtorch to allow epitaxial reconstruction to large Au(111) terraces. Tips were cut from Au wire (0.25 mm, annealed) purchased from Goodfellow Cambridge Ltd. The experiments were performed in liquid environment (mesitylene 98 %, Sigma-Aldrich) in the presence of the target molecule in 1 mM concentration. We used a modified commercial STM (Keysight Technologies 5500 SPM) for the measurement. Signal are routed in and out of the STM controller through a break-out box (Keysight Technologies N9447A). Piezo and voltage signal are imposed to the STM by an arbitrary waveform generator (Keysight Technologies 33522B). The current signal is converted to voltage by a transimpedance amplifier (Femto GmbH DLPCA-200) and all signals are then acquired by a National Instruments PXI system (PXIe-1062Q chassis with a PXIe-4464 24-bit acquisition module and PXIe-PCIe8381 interface). Data acquisition and processing is performed with Labview. Data is acquired at 20 kHz for low-frequency modulation, and at 200 kHz for high-speed experiments.

In a typical experiment, a fixed bias is applied to the junction (200 mV in this study), and the Au tip is driven into the sample until the conductance of the junction is >5 G_0, and then abruptly retracted of 1 nm. The desired modulation is then applied for 100 ms, and the junction is stretched again. Thousands of consecutive traces are collected, and these are then sliced between the stretches by analyzing the signal imposed to the piezo actuator and cutting where its second derivative is above 0.1.

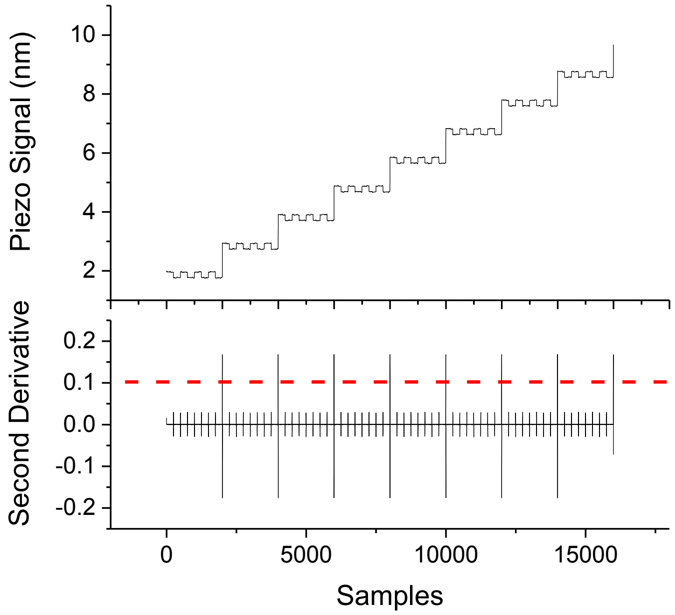

Figure S5: Example of piezo signal of a modulation experiment (in nm) and its second derivative, which is used to slice a single trace into individual modulation “snippets”. Second derivative threshold is shown as red dashed line.

The transimpedance amplifier signal is converted to current by applying the conversion factor (10^6 or 10^5 V/A, depending on the bandwidth and sensitivity needed), and then conductance is determined by Ohm's law ($G = I/V$) and divided by the quantum of conductance G_0 (≈ 77.48 µS). The individual slices are then fed into a sorting algorithm, which takes the average of the conductance of the first and last modulation and checks that both are below 0.1 G_0 and above the noise level of the preamplifier (10^{-5} G_0). This filters out the slices where the tip is in contact or shallow interaction with the substrate, those where no molecular bridge is present, and those where the molecular bridge did not survive the whole modulation process, leaving only the slices relative to molecular junctions.

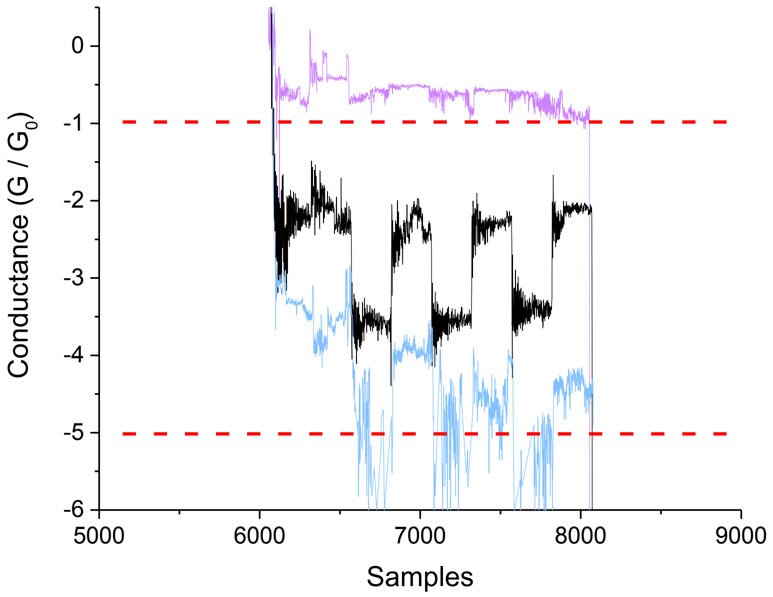

Figure S6: Examples of traces fed into the sorting algorithm. Traces above 10^{-1} G_0 (purple) or below 10^{-5} G_0 (blue) are filtered out, and only traces with stable molecular junctions (black) are kept. Conductance boundaries are shown as red dashed lines.

All the slices which are selected by the automated algorithm are then used to compile the density maps presented in the manuscript and here in the SI in section 3.

In absence of a molecular wire in solution, the conductance traces show a clear breaking of the Au-Au contact with abrupt decay to conductance values very close to the noise level of the instrument. Modulation of gap size after the 1 nm stretch resulted in changes of conductance in the range $10^{-4.5} – 10^{-6.5}$ G_0.

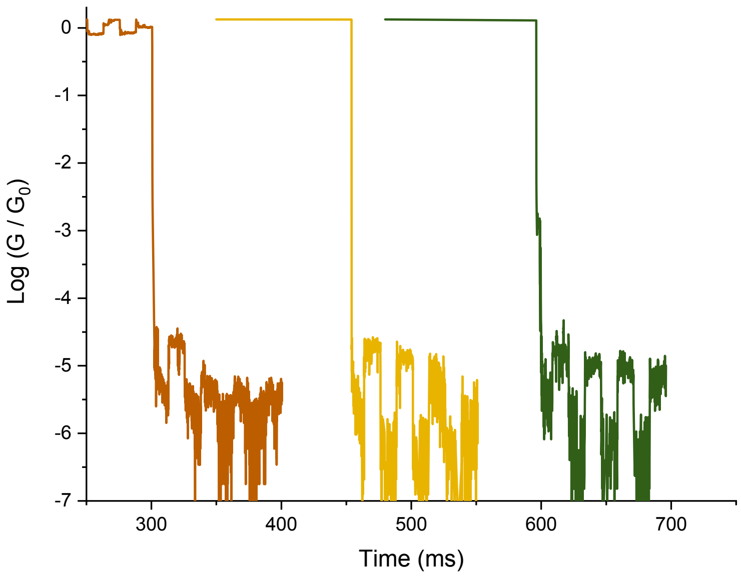

Figure S7: Example modulation traces obtained in pure mesitylene.

3. Additional single-molecule conductance data

***STM-BJ* data**

As discussed in the introduction, a large spread of conductance values is generally found in oligothiophene-based molecular wires, and we focussed our investigation on the bridged bithiophenes **1** and **2** which were found to result in sharper conductance histogram peaks.[6] However, while measurements on compound **1** indeed produces sharper histograms than measurements on 5,5'-bis(methylthio)-2,2'-bithiophene, the span of conductance values is still significantly larger than in the simple biphenyl **3** (Figure S8).

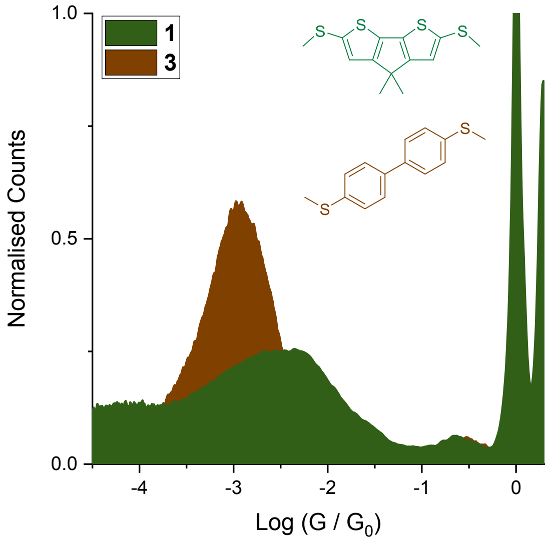

Figure S8: Conductance histograms for compounds **1** and **3**. Both recorded at 200 mV bias, >5000 scans each (data normalised to the number of scans used to compile the histograms)

Furthermore, analysing the single-molecule conductance traces, it was evident that while in compound **3** conductance reliably settles on a plateau as the tip is retracted, traces for compounds incorporating a thienyl moiety fail to do so (Figure S8). In *STM-BJ* conductance traces for **1** or **2**, plateau-like features are either absent, heavily sloped, or even extending well into the noise level of our preamplifier (Figure S9), a behaviour we attributed to the continuous sampling of different possible molecule-electrode interactions as the two electrodes are pulled apart during an *STM-BJ* measurement.

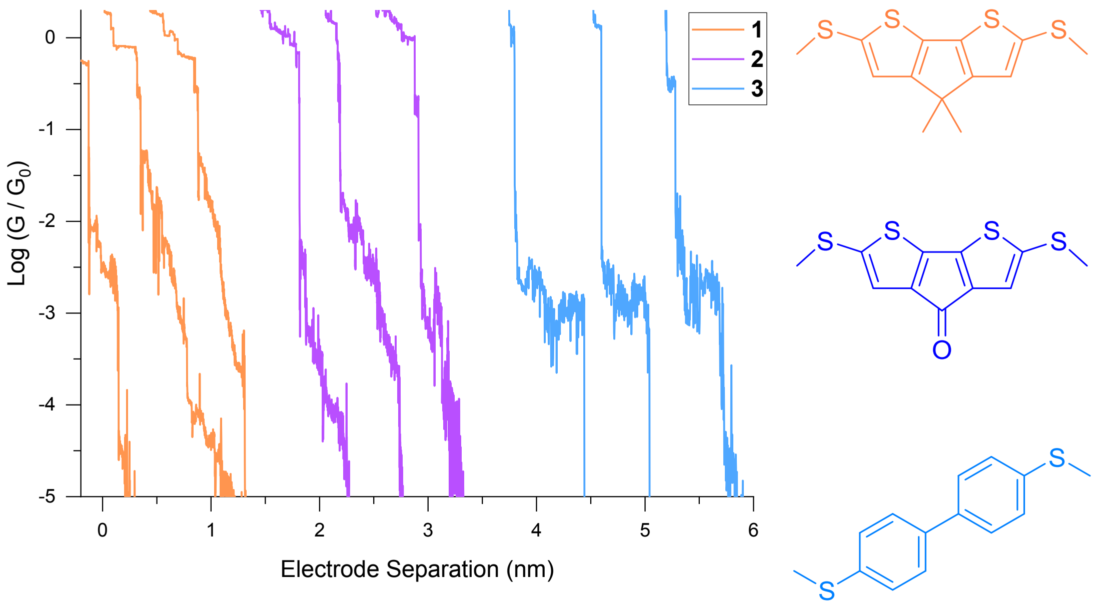

Figure S9: Example *STM-BJ* traces for compound **1**, **2** and **3**. Traces recorded at 100 mV tip bias.

Additional Piezo-Modulation Data

In addition to the 3 Å modulation presented in the manuscript, we recorded data with a smaller (2 Å) and a larger (4 Å) amplitude. Results are presented here, as 2D density maps.

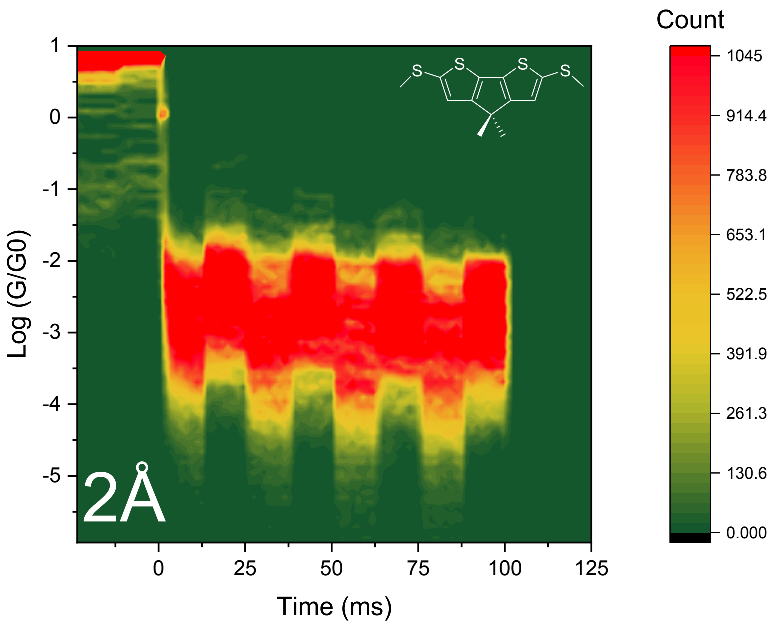

Figure S10: Density map for compound **1** under 2 Å square modulation. The plot is normalised to the number of scans used to compile it (1045).

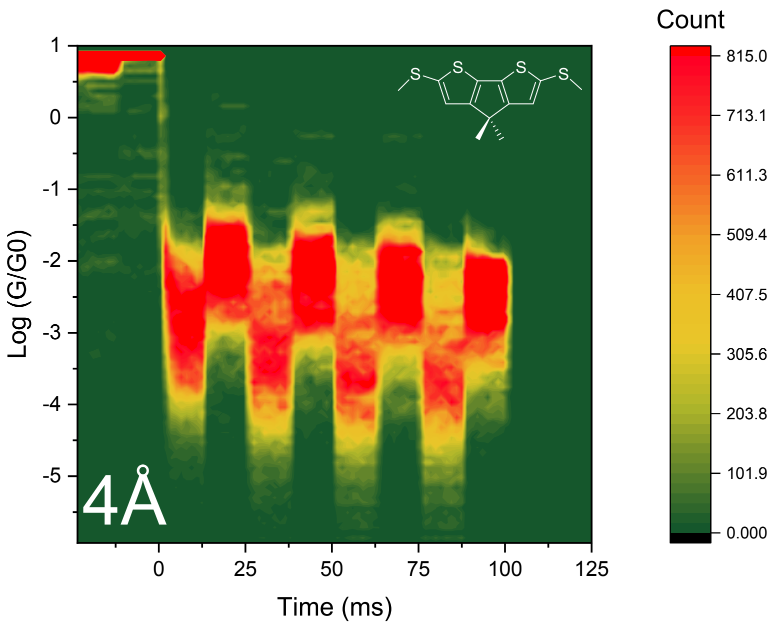

Figure S11: Density map for compound **1** under 4 Å square modulation. The plot is normalised to the number of scans used to compile it (815).

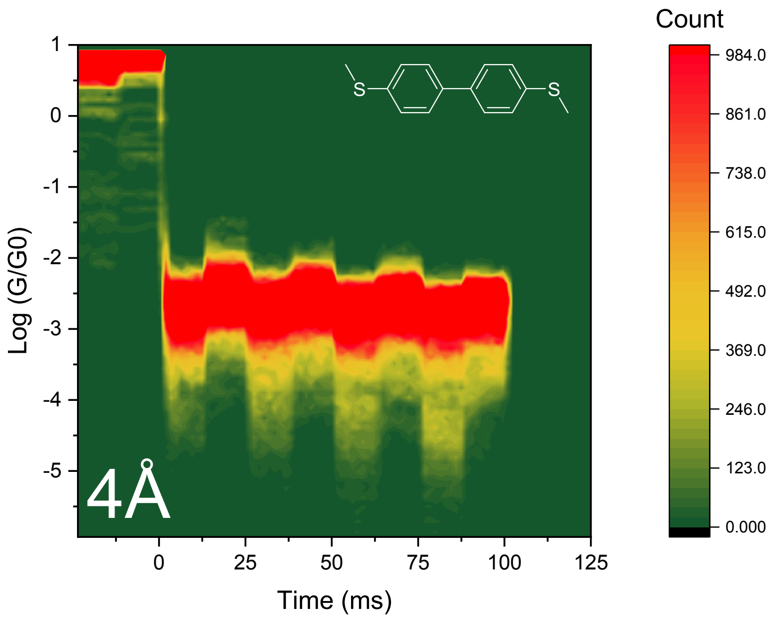

Figure S12: Density map for compound **3** under 4 Å square modulation. The plot is normalised to the number of scans used to compile it (984).

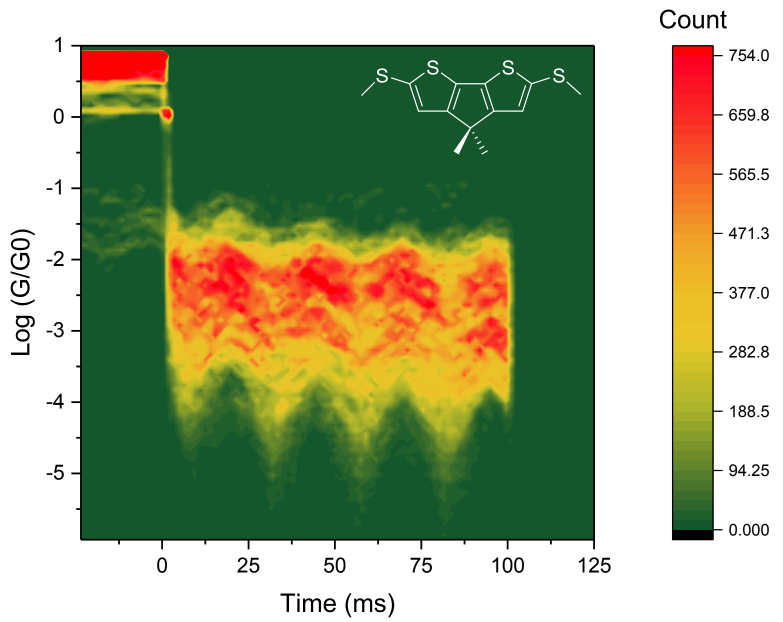

Figure S13: Density map for compound **1** under 3 Å triangular modulation. The plot is normalised to the number of scans used to compile it (754).

In the main paper, we presented only a 2 ms "snippet" of the 10 kHz modulation trace. Further data is presented here.

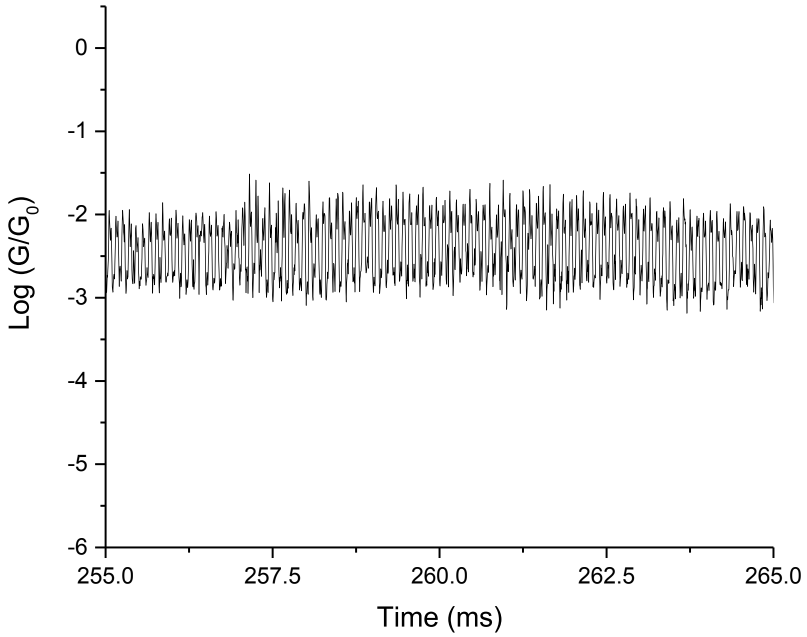

Figure S14: 10 ms of an example trace of high-speed (10 kHz) square-wave modulation of compound **1**

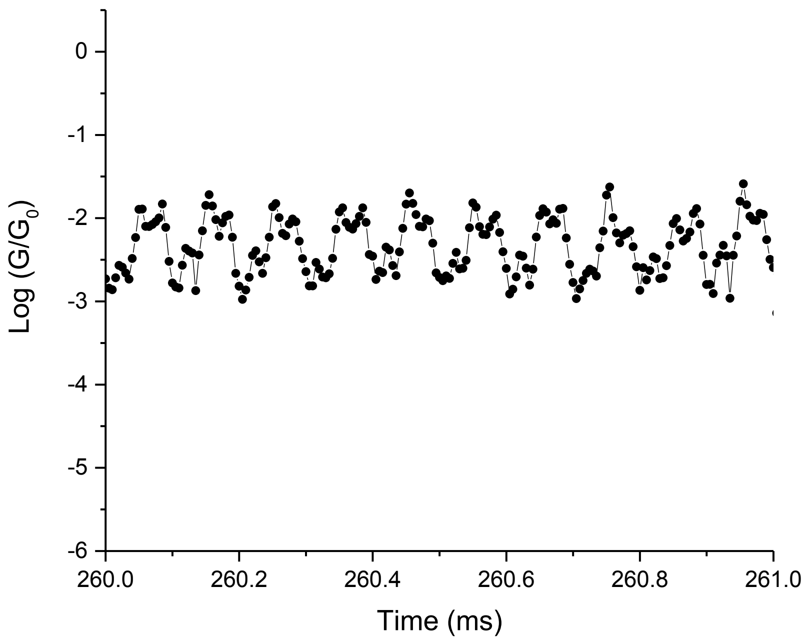

Figure S15: 1 ms zoom of Figure S14 (10 kHz square-wave modulation of compound **1**)

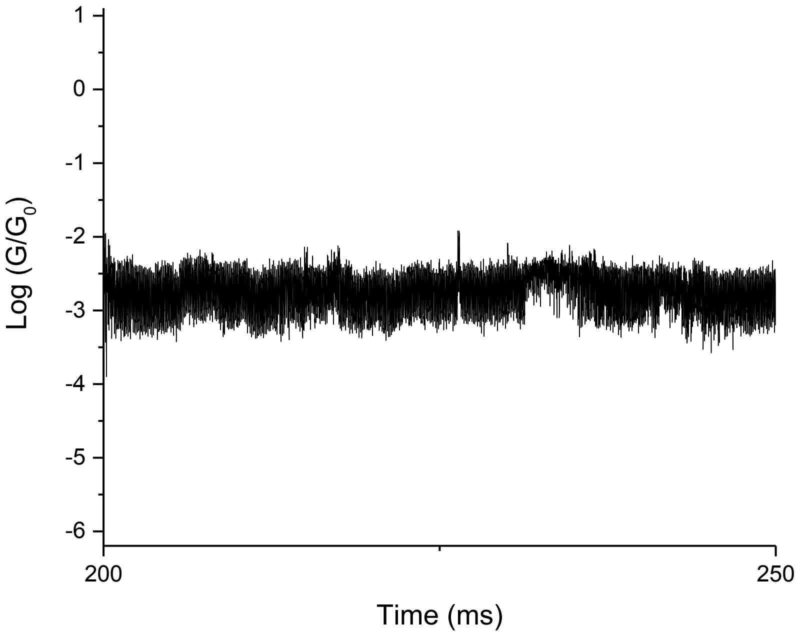

Figure S16: Example of a full 50 ms high-speed (10 kHz) square-wave modulation of compound **1**

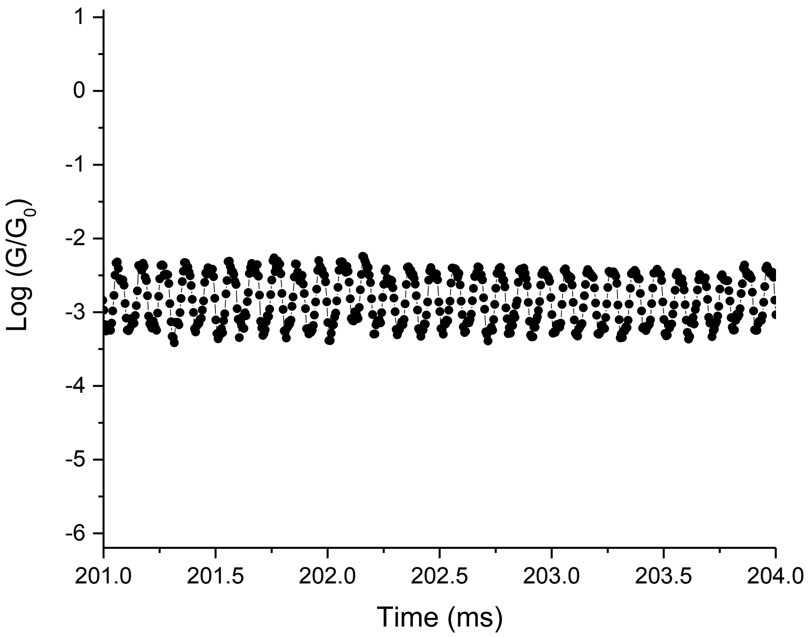

Figure S17: 3 ms zoom of Figure S16 (10 kHz square-wave modulation of compound **1**)

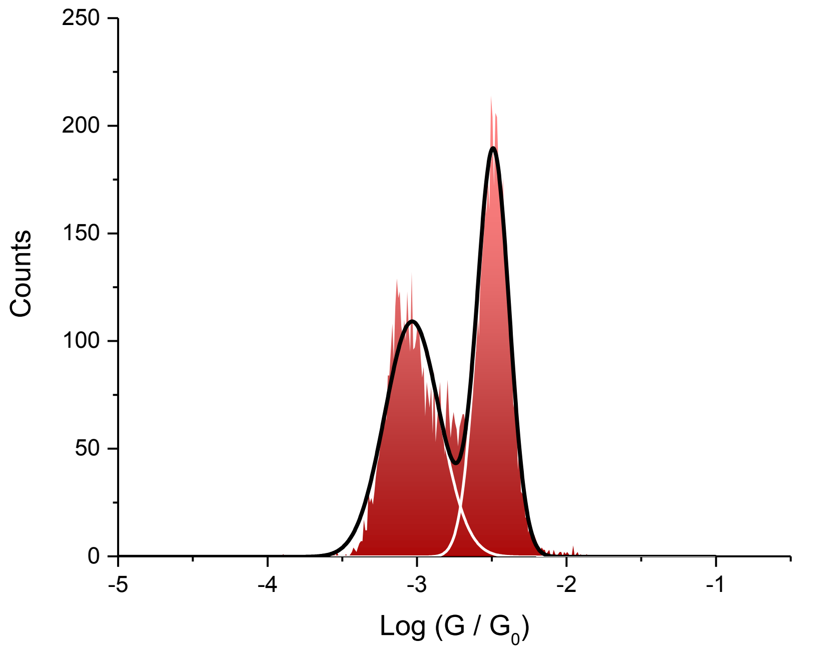

Figure S18: Histogram (100 bins per decade) for the full 50 ms high-speed modulation trace shown in Figure S16. The peak heights for the compressed and relaxed junction are different, but the integrated area of the two gaussian fittings is equivalent (49.7 and 50.1). This data is in good agreement with the histograms presented for the full low-speed dataset in Figure 3 of the main text.

We also performed experiments on **4** at 2 Å square-wave modulation. The compound shows slightly reduced modulation amplitude, but still a clear HIGH-LOW conductance switching.

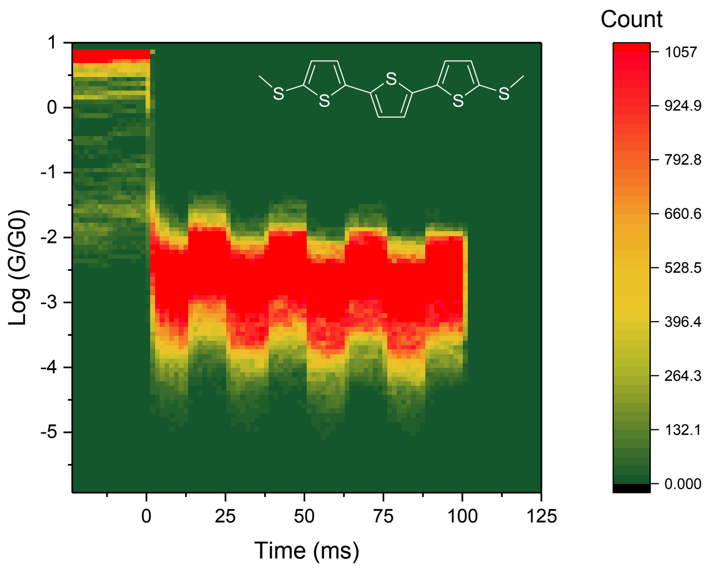

Figure S19: Density map for the terthiophene compound under 2 Å square-wave modulation. Structure is superimposed on the map. The plot is normalised to the number of scans used to compile it (1057).

4. Details on DFT Methods and Additional Calculations

The transmission coefficient *T(E)* for electrons of energy *E* (passing from the source to the drain) is calculated via the relation: $T(E) = Trace\left(\Gamma_R(E)G^R(E)\Gamma_L(E)G^{R\dagger}(E)\right)$. In this expression, $\Gamma_{L,R}(E) = i\left(\sum_{L,R}(E) - \sum_{L,R}{}^{\dagger}(E)\right)$ describe the level broadening due to the coupling between left (L) and right (R) electrodes and the central scattering region, $\sum_{L,R}(E)$ are the retarded self-energies associated with this coupling and $G^R = (ES - H - \sum_L - \sum_R)^{-1}$ is the retarded Green's function, where H is the Hamiltonian and S is overlap matrix. Using obtained transmission coefficient $T(E)$, the conductance could be calculated by Landauer formula ($G = G_0 \int dE\, T(E)(-\partial f/\partial E)$) where $G_0 = 2e^2/h$ is conductance quantum and $f = 1/[1 + \exp(E - E_F)/k_B T]$, where E_F is the Fermi energy. If $T(E)$ veries slowly of the scale of $k_B T$ at room temperature T, then $G = G_0 T(E_F)$.

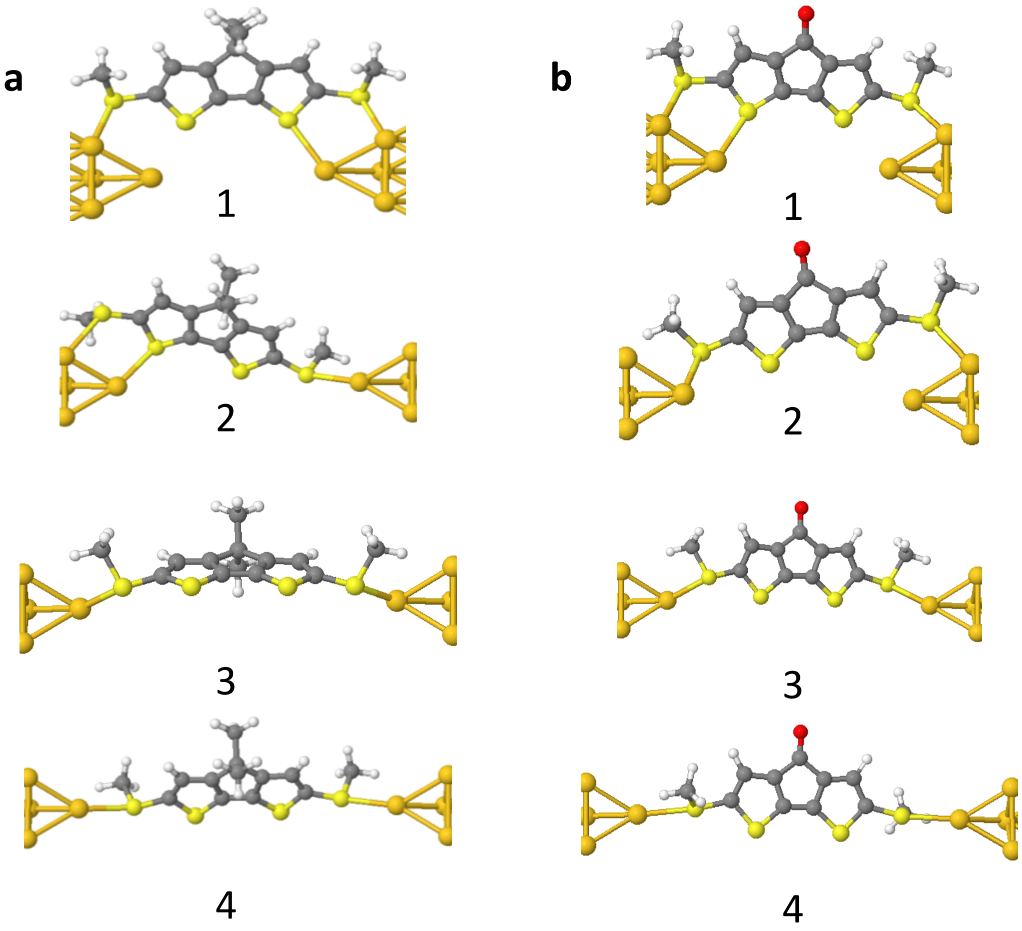

Figure S20: Relaxed structure of molecule **1** (a) and **2** (b) in a junction where the tip to tip distances is 7 Å for junction 1 and is stretched by ~ 2 Å at any step for junction 2-4.

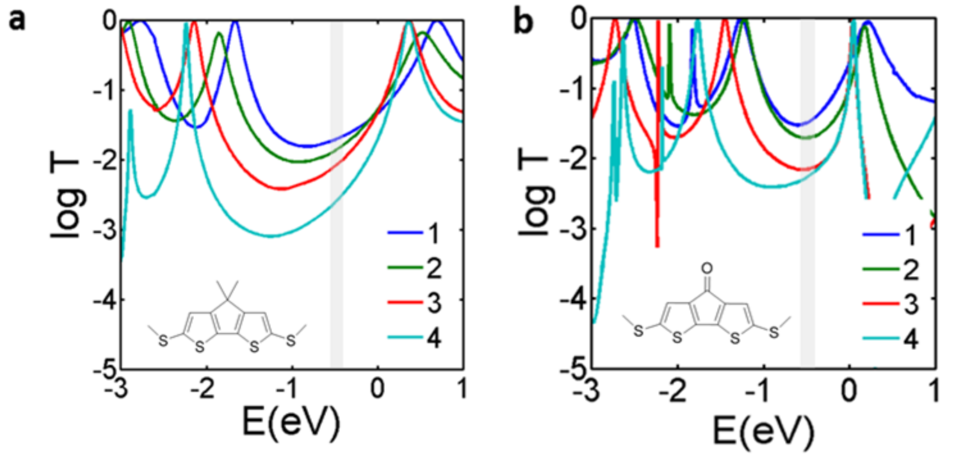

Figure S21: Transmission coefficient of compounds **1** (a) and **2** (b)**,** calculated from the structures shown in Figure S20. The grey region shows the Fermi energy where the amplitude of conductance is in agreement with measured values.

A single Breit-Wigner resonance, using the formula $T(E_F) = \frac{4\Gamma^2}{(E_F-\varepsilon-\Sigma)^2+4\Gamma^2}$, was then fitted to the transmission curves presented here and in the manuscript, using the maximum of the LUMO resonance (the closer to E_F) as the value of $(\varepsilon - \Sigma)$, therefore parametrising the value of Γ. An example for compound **1** can be found in Figure S22.

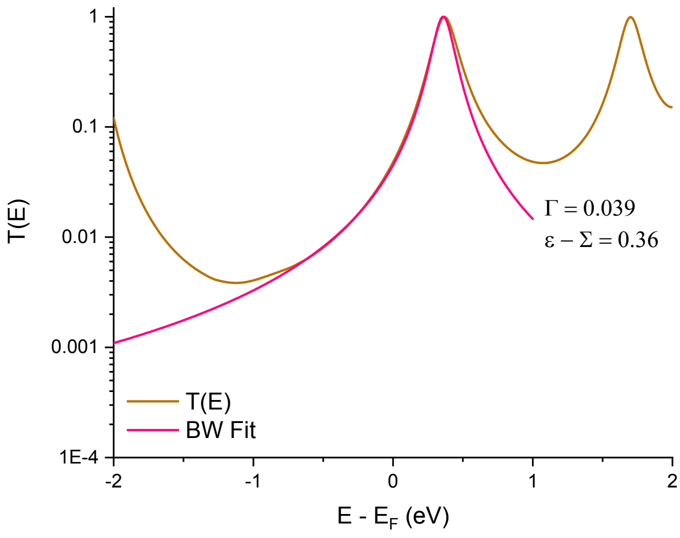

Figure S22: Example of single resonance fitting to obtain values for Γ. Shown here, compound **1**, DFT relaxed structure with electrode separation of 13.41 Å

The results are summarised in Figure S23. As discussed in the manuscript, a clear pattern of increasing Γ as the electrodes are compressed can be found in compounds **1, 2** and **4**, but no pattern was found in **3**.

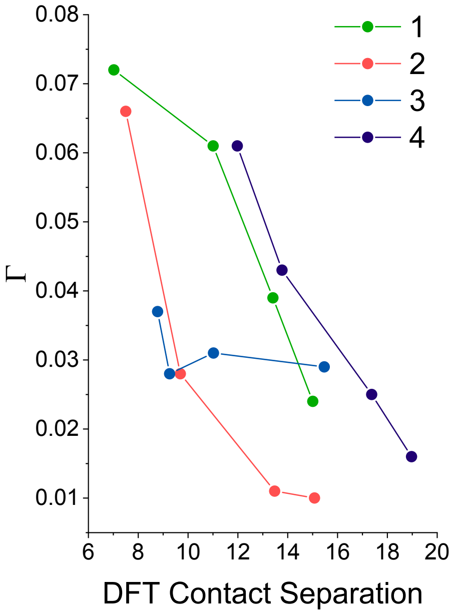

Compound	Electrode Separation (Å)	Γ
**1**	7.02	0.072
	11.01	0.061
	13.41	0.039
	15.01	0.024
**2**	7.49	0.066
	9.69	0.028
	13.48	0.011
	15.08	0.01
**3**	8.78	0.037
	9.26	0.028
	11.02	0.031
	15.47	0.029
**4**	11.97	0.061
	13.77	0.043
	17.37	0.039
	18.97	0.024

Figure S23: Calculated Γ for compounds **1-4** at different electrode separation. Plot (left) and numerical values (right).

5. References

1. Williams, D. B. G. & Lawton, M. Drying of organic solvents: quantitative evaluation of the efficiency of several desiccants. *J. Org. Chem.* **75**, 8351–4 (2010).

2. Burchat, A. F., Chong, J. M. M. & Nielsen, N. Titration of alkyllithiums with a simple reagent to a blue endpoint. *J. Organomet. Chem.* **542**, 281–283 (1997).

3. Smeets, B. J. J., Meijer, R. H., Meuldijk, J., Vekemans, A. J. M. & Hulshof, L. A. Process Design and Scale-Up of the Synthesis of 2,2′:5′,2″-Terthienyl. *Org. Process Res. Dev.* **7**, 10–16 (2003).

4. Yan, P., Xie, A., Wei, M. & Loew, L. M. Amino(oligo)thiophene-Based Environmentally Sensitive Biomembrane Chromophores. *J. Org. Chem.* **73**, 6587–6594 (2008).

5. Hanamura, H. & Nemoto, N. Synthesis and properties of cyclopentadithiophene-based poly(silarylenesiloxane) derivatives. *Polymer (Guildf).* **52**, 5282–5289 (2011).

6. Dell, E. J. *et al.* Impact of molecular symmetry on single-molecule conductance. *J. Am. Chem. Soc.* **135**, 11724–7 (2013).
